# Knowledge, Attitude and Worry in the Kurdistan Region of Iraq during the Mpox (Monkeypox) Outbreak in 2022: An Online Cross-Sectional Study

**DOI:** 10.3390/vaccines11030610

**Published:** 2023-03-08

**Authors:** Sirwan Khalid Ahmed, Salar Omar Abdulqadir, Rukhsar Muhammad Omar, Ardalan Jabbar Abdullah, Hawre Asaad Rahman, Safin Hassan Hussein, Halkawt Ismail Mohammed Amin, Deepak Chandran, Anil Kumar Sharma, Kuldeep Dhama, Malik Sallam, Harapan Harapan, Nader Salari, Chiranjib Chakraborty, Araz Qadir Abdulla

**Affiliations:** 1 Department of Pediatrics, Rania Pediatric & Maternity Teaching Hospital, Rania, Sulaymaniyah 46012, Iraq; 2 Department of Nursing, University of Raparin, Rania, Sulaymaniyah 46012, Iraq; 3 Department of Kindergarten, College of Basic Education, University of Raparin, Rania, Sulaymaniyah 46012, Iraq; 4 Department of Emergency Nursing, Hibat Sultan Technical Institute, Koye, Erbil 46017, Iraq; 5 Department of Business Information Technology, Haibat Sultan Technical Institute, Koya, Erbil 46017, Iraq; 6 Department of Medical Laboratory Science, College of Science, University of Raparin, Rania, Sulaymaniyah 46012, Iraq; 7 Department of Accounting, Dukan Technical Institute, Sulaimani Polytechnic University, Sulaymaniyah 46010, Iraq; 8 Department of Veterinary Sciences and Animal Husbandry, Amrita School of Agricultural Sciences, Amrita Vishwa Vidyapeetham University, Coimbatore 642109, Tamil Nadu, India; 9 Department of Biotechnology, Maharishi Markandeshwar University Deemed to Be University, Mullana-Ambala 133207, Haryana, India; 10 Division of Pathology, ICAR-Indian Veterinary Research Institute (IVRI), Izatnagar, Bareilly 243122, Uttar Pradesh, India; 11 Department of Pathology, Microbiology and Forensic Medicine, School of Medicine, The University of Jordan, Amman 11942, Jordan; 12 Department of Clinical Laboratories and Forensic Medicine, Jordan University Hospital, Amman 11942, Jordan; 13 Department of Translational Medicine, Faculty of Medicine, Lund University, 22184 Malmö, Sweden; 14 Medical Research Unit, School of Medicine, Universitas Syiah Kuala, Banda Aceh 23111, Indonesia; 15 Tropical Diseases Centre, School of Medicine, Universitas Syiah Kuala, Banda Aceh 23111, Indonesia; 16 Department of Microbiology, School of Medicine, Universitas Syiah Kuala, Banda Aceh 23111, Indonesia; 17 Department of Biostatistics, Faculty of Public Health, Kermanshah University of Medical Sciences, Kermanshah 6715847141, Iran; 18 Department of Biotechnology, School of Life Science and Biotechnology, Adamas University, Kolkata 700126, West Bengal, India

**Keywords:** monkeypox, mpox, knowledge, attitude, worry, vaccine acceptance, social media, fear, stress, vaccine hesitancy

## Abstract

The rapid spread of monkeypox (mpox) has been declared as a public health emergency of international concern (PHEIC). The present study aimed to assess the knowledge, attitude, and worry levels of the general population in the Kurdistan region of Iraq regarding the ongoing mpox multi-country outbreak. An online cross-sectional survey was conducted between 27–30 July 2022, using a convenience sampling method. The questionnaire was adapted from previous studies addressing the same topic. The independent Student’s *t*-test, one-way ANOVA, and logistic regression were used to assess possible factors associated with knowledge, attitude, and worry toward mpox. A total of 510 respondents were included in the final analysis. The participants showed a moderate level of mpox knowledge, a neutral attitude towards mpox, and a relatively moderate worry level. The logistic regression analysis showed that age, gender, marital status, religion, level of education, and place of residence were associated with mpox knowledge; however, the significant variables in the multivariate regression analysis were gender, religion, level of education, and residential area. Gender and residential area were associated with attitudes toward mpox; however, the significant variables in the multivariate regression analysis were gender and residential areas. The worry toward mpox was influenced by gender, marital status, religion, and place of residence, yet the significant variables in the multivariate regression analysis were gender, religion, educational level, and residential area. In conclusion, the Kurdish population had moderate knowledge, a neutral attitude, and a moderate level of worry about mpox. Considering the continuous rapid rise in mpox cases in several countries, and its possible risk as pandemic amid the ongoing COVID-19 pandemic, proactive control measures, adequate disease prevention strategies, and preparedness plans need to be formulated and immediately implemented to tackle the appearance of fears among people, and to safeguard the mental health of the public.

## 1. Introduction

Against the backdrop of the ravages of the coronavirus disease 2019 (COVID-19) pandemic, the recent resurgence of the monkeypox (mpox) outbreak caused by the monkeypox virus (MPXV) has raised concerns about the prospect of another global outbreak [1,2]. The global spread of mpox in more than 110 countries/territories worldwide has been reported in people who have no proven travel ties to regions previously endemic for mpox [3,4,5,6]. The mpox cases rose rapidly, leading to its declaration as a public health emergency of international concern (PHEIC) on 23 July 2022 by the World Health Organization (WHO) [2,7,8,9]. As of 27 January 2023, a total of 85,142 laboratory-confirmed mpox cases were recorded globally, with 86 deaths from the disease [10].

As a zoonotic disease, mpox has been previously reported in several parts of the world, particularly in western and central Africa, which are endemic for mpox, and the disease has often been considered a neglected tropical disease [2,3,4,5,6]. Mpox was first reported in a patient in the Democratic Republic of the Congo (DRC) who had symptoms like of smallpox; the virus was officially recognized as a separate human infection via zoonosis [2]. Later, other human mpox cases with zoonotic infections proved animal spillover of MPXV to humans [2]. The incubation period for MPXV ranges between 5 and 21 days, and the virus spreads by droplets and through contact with skin lesions or contaminated bodily fluids and materials of the infected people [2,11]. In addition, MPXV transmission can occur via the vertical pathway (i.e., mother-to-child transmission) [12]. Furthermore, sexual transmission of MPXV was notable during the ongoing mpox outbreak [2,13]. Rodents are among the proposed reservoirs for MPXV, and exposure factors include coming into contact with animals (living or dead) and consuming bush foods [2,4,14].

The symptoms of mpox are self-limiting and typically disappear after 14–21 days [15]. Mild-to-severe manifestations of mpox include fever, headache, lethargy, lymphadenopathy, back pain, and myalgia, in addition to the itchy to painful skin lesions that are the disease’s hallmark [2,15]. The skin rash that might occur up to three days after the fever has subsided is the most prominent clinical symptom [2,16]. It typically starts off looking like a maculopapular eruption, then turns into vesicles, pustules, and finally crusts. It first manifests on the face, then spreads rapidly to the rest of the body and extremities, and can eventually affect the entire body in extreme cases. Compared with smallpox, the most notable distinction is that mpox leads to lymphadenopathy [2,15,17]. In certain cases, the skin lesions might remain for up to four weeks [18].

The rising incidences of human mpox highlights the need for vigilant surveillance for the disease, prompt diagnosis, and developing effective vaccines, drugs, and treatment options [19,20,21]. Primary prevention of mpox is achieved by public awareness campaigns that highlight the risk factors for the disease and the preventative steps that can be taken to lessen those risks [15,22,23]. However, an early report by the WHO revealed that a lack of knowledge regarding mpox, particularly among healthcare providers, can be a major barrier to the preventive efforts [24]. Previous studies have found that the knowledge level about mpox is relatively low among health professionals and students as well as among the public in various regions of world, including the Middle East [25,26,27,28,29,30,31,32,33]. In the first month following the WHO alert, public concern over COVID-19 outpaced that of mpox in some countries outside Africa [34]. 

In addition, worry and stress are typical reactions to infectious illness epidemics such as mpox [35]. The public’s uncertainty, concern, panic, and fear are understandable given that mpox is a re-emerging disease that could cause severe consequences worldwide [35]. Misconceptions and false information about mpox have also spread rapidly in the era of broad social media use [35]. As a result, everyone is feeling the effects of widespread worry to varying degrees. The public’s awareness and attitude are hypothesized to have a significant impact on the mental health and clinical outcomes. For this reason, researching these facets in different communities is crucial. Another important health risk that is projected to grow steadily and worsen during this epidemic is that of mental disorders. Despite the public’s widespread fear and panic during the mpox outbreak, patients, survivors, and their families face significant stigma and social isolation [36,37].

In light of the aforementioned information, the current study was designed to measure general population knowledge regarding mpox disease as a part of prevention and health promotion and to raise knowledge, attitude, and to reduce the level of worry about the present epidemic. Furthermore, in nations with a weak healthcare system, assessing the association between disease knowledge and attitude can have beneficial effects on comprehending health-seeking behavior and observing preventative measures [38]. The resurgence and global spread of mpox represent a significant risk to human health and safety, particularly in non-endemic countries such as Iraq. Therefore, this study sought to assess and analyse the general population’s knowledge, attitudes, and worry about mpox in the Kurdistan region of Iraq.

## 2. Methods

### 2.1. Study Design, Setting, Period, and Sample Size

This was an online cross-sectional study conducted in all provinces in the Kurdistan region of Iraq (Sulaymaniyah, Erbil, Duhok, and Halabja). Participants living in those provinces, aged 18 years or older, and understanding the content of the questionnaires were prompted to fill out the survey. The convenience sampling method was used to collect the data between 27–30 July 2022. The sample size was computed using the confidence interval at 95%, with an estimated 50% response distribution and a margin of error of 5%. The required minimum sample size was determined to be 382 individuals, with a total population of Kurdistan-region was 5.2 million.

In order to guarantee a large-scale distribution and recruitment of participants, the questionnaire was administered in an online format using Google Forms, and the link of the survey was distributed via Facebook, WhatsApp, Twitter, and email lists. 

### 2.2. Study Tool

The questionnaire was adapted from prior published studies on mpox and COVID-19 [28,33,39], and modified to suit this study (Supplementary File S1). Various adjustments were made according to the suggestions made by the virologist and psychiatrists. To guarantee accurate results, the questions were translated from English to Kurdish using a forward-backwards method. A total of 5 minutes were needed to complete the questionnaire.

The questionnaire was divided into 4 main parts. The first part gathered information about the sociodemographic characteristics of respondents, including age, gender, marital status, religion, educational attainment, and the geographic location. The second section assessed knowledge about mpox related to modes of transmission, isolation, prevention, treatment, and symptoms. This section consisted of 8 items. The third part of the questionnaire consisted of 4 questions related to the attitude toward mpox. This section includes 4 items. The fourth part included questions related to the worry about MPXV infection and assessed using 9 items.

Before the study questionnaire was used in the actual study, it was tested in the initial study to assess the internal consistency of the items among 13 participants of general population on 27 July 2022. The data of this initial study were excluded from the final analysis. Cronbach’s alpha was used to calculate the internal consistency of the items. The Cronbach’s alpha for knowledge, attitude, and worry scales were 0.72, 069, and 0.90, respectively with overall Cronbach’s alpha for the entire questionnaire was 0.80, which indicates acceptable internal consistency [40].

### 2.3. Study Variables

The response variables of this study were knowledge, attitude, and worry about mpox. Knowledge on mpox was assessed using eight items: three questions with answer options “yes” and “no” of which score of 1 was provided for “yes” and 0 for “no”; two questions were on a Likert-point scale (“true”, “false”, and “I do not know”) of which score 1 for “yes” and 0 for “I do not know” and “no” responses; and three questions were checkboxes to choose the correct answers of which score 1 was given to each correct answer. The total score ranged between 4 and 14. The scores were then divided into three categories: poor (score 4–5), fair (score 6–7), and good (score 8–14). 

Attitude toward mpox was assessed using four items. Participants were asked to rate attitude items as either “true” or “false” with an additional “I do not know” option. The score of 1 was given for “true” and 0 for “false” and “I do not know” selections. Therefore, the total score ranged between 0 and 4, of which the higher score indicated a positive attitude. The score was divided into negative attitude (score 0–1), neutral attitude (score 1.1–2), and positive attitude (score 2.1–4). 

Worry about MPXV infection was assessed using nine items with a five-point Likert scale ranging from “never”, “rarely”, “sometimes”, “often”, and “always”. The score 5 was given for “always” and 1 for “never”. The total score ranged between 5 and 45; of which the score 5 to 25 was classified as low level of worry, score 25.1 to 40 as moderate level, and the score 41.1 to 45 as a high level of worry. 

Some explanatory variables were measured during the study, including age, gender, marital status, religion, educational attainment, and the geographic location. Age was divided into four categories (18–27, 28–37, 38–47, and 48–57-year-old). The religion was divided into Muslims, Christians, and others, and the marital status was recorded as single and married. Educational attainment was categorized into being able to read and write, middle and/or high school, bachelor’s degree, and postgraduate degree.

### 2.4. Ethical Approval and Inform Consent

This study followed the Institutional Research Ethics Board and the Declaration of Helsinki guidelines. The Ethical Review Committee of the University of Raparin College of Nursing approved this study (Ref: 7/29/408). Electronic informed consent was obtained from all participants before filling out the questionnaires. On the first page of the survey, the study aims and benefits were provided to all participants, and privacy and confidentiality of the information were also ensured.

### 2.5. Statistical Analysis

Percentages and frequencies were used to describe categorical data, whereas mean and standard deviation were used to characterize continuous variables. The Kolmogorov–Smirnov test was used to evaluate the normal distribution of variables studied, and none of the variables followed the normal distribution. Furthermore, to test the normality of the data, the standard error of the skewness coefficient and the standard error of the kurtosis coefficient were used, according to the central limit theorem, considering the large size of the research sample (*n* = 510) and that the standard error of the skewness and kurtosis between (2+ and −2); therefore, the data distribution is normal and parametric tests were used: independent Student’s *t*-test in the case of two groups, and the one-way ANOVA test for three groups or more. The dependent variables consisted of three possible outcomes; therefore, univariate and multivariate logistic regression analysis was used to assess the predictive factors for good knowledge, a positive attitude, and a high level of worry toward mpox cut-off points. All variables that had *p* < 0.25 in univariate logistic regression analysis were retained for the multivariate logistic analysis. For each independent variable, the crude odds ratio (OR) and 95% confidence interval (CI) were initially computed in the univariate logistic regression analysis; then, the adjusted odds ratio (aOR) and 95% confidence interval (CI) were computed in the multivariate logistic regression analysis. The *p* value was set at <0.05 for statistical significance. 

## 3. Results

### 3.1. Sociodemographic Characteristics

A total of 510 responses were analyzed. Responses to all survey questions were required for successful submission of the questionnaire. None of the participants were excluded. The ages ranged between 18 and 57 years (Table 1). The participants in the study were limited to only individuals who had access to the internet. Approximately 40% of participants were holders of bachelor’s degrees, with a mean age of 31.1 ± 8.5 years-old (Table 1). Moreover, 54.3% males and 45.7% were females. The participants originated from four different provinces in the Kurdistan-region of Iraq, with the most participants from Sulaymaniyah (50%), followed by Erbil (23%), Duhok (13.8%), and Halabja (13.2%). Approximately 94.5% of the participants were Muslim (Table 1).

### 3.2. Response Distribution of Knowledge about Mpox

**Figure 1:**
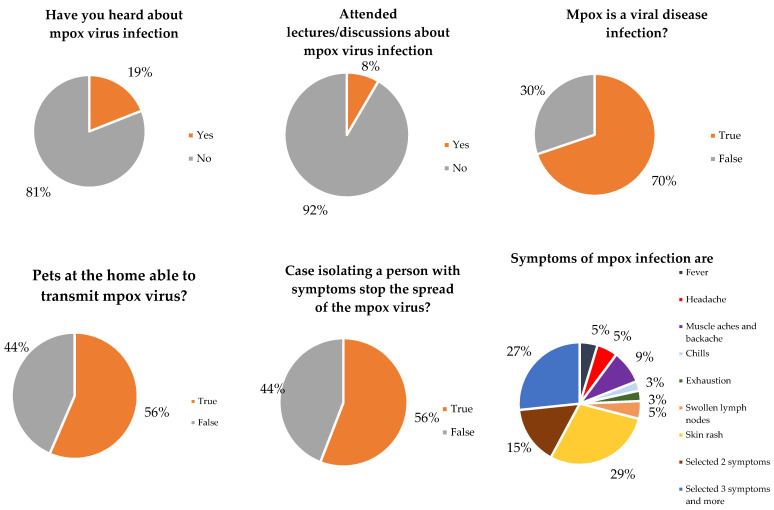

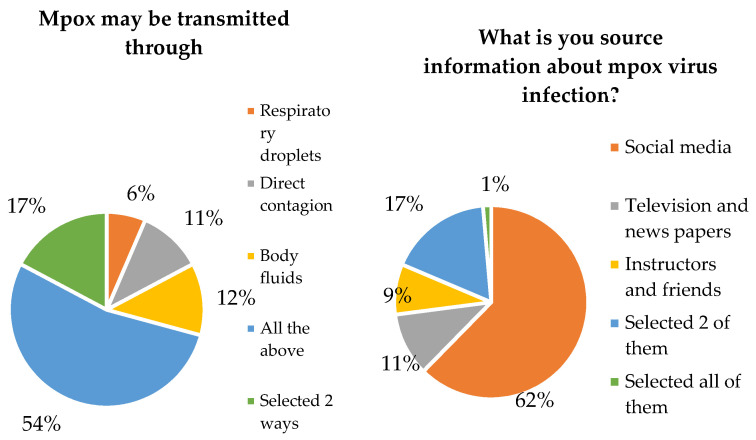
Knowledge of participants about mpox viral infection (*n* = 510).

Our data suggested that 70% of participants believed that mpox is a viral infectious disease; 81% never heard of mpox, 92% never attended a lecture or discussion about mpox, and 56% believed that isolating people with symptoms of the disease will stop the spread of the epidemic (Figure 1). Furthermore, 54% of participants believed that the virus would be transmitted in more than one way, 26.7% of the participants chose more than one item as a symptom of the disease, and 62.2% selected social media as a source of their knowledge of the disease (Figure 1).

### 3.3. Response Distribution of Attitude toward Mpox

**Figure 2:**
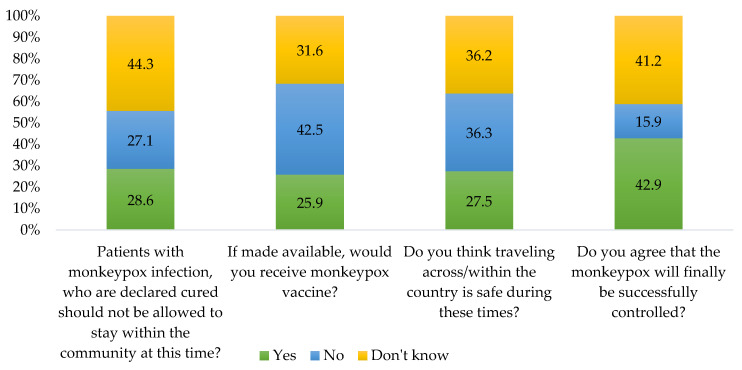
Attitude of participants about mpox viral infection (*n* = 510).

Our data suggested that 44.3% of participants did not have any idea about whether a mpox patient should stay at home or not, and 42.5% of them mentioned that they will refuse vaccination for mpox (Figure 2). In addition, 36.3% of the participants did not agree with travelling across the country is safe during the mpox outbreak, and 42.9% of the participants agreed with the idea that the mpox epidemic will be successfully controlled (Figure 2).

### 3.4. Response Distribution of Worry toward the Mpox Outbreak

**Figure 3:**
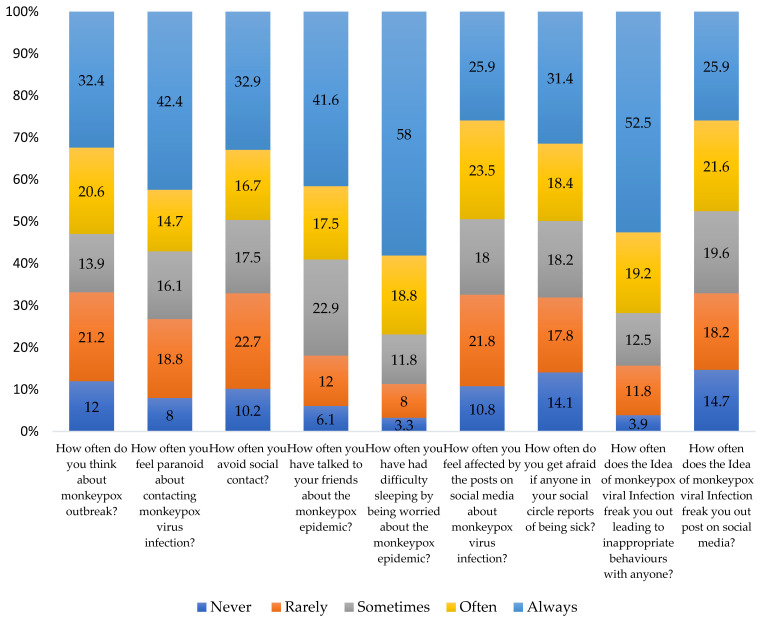
Worry related mpox outbreak in 2022 (*n* = 510).

The worry level of participants about the current mpox outbreak is presented in (Figure 3). In the preceding two weeks, over half of the participants had been preoccupied by the mpox outbreak, ranging from sometimes to always. The fear of contracting the MPXV caused paranoia in 43% of the respondents, and 41% admitted to feeling terrified when a friend or a family member became sick, ranging from sometimes to always. However, 11% of the participants reported having difficulty in sleeping in the past two weeks owing to worry about the current outbreak. The frequency of these difficulties ranged from often to always. Furthermore, 50% of the respondents had restricted social connections, and 42% never discussed the outbreak with their friends. Approximately 16% of participants were engaged in inappropriate social behavior due to their fear of contracting the virus. The frequency of these behaviors ranged from often to always. Almost 26% of participants never posted anything on social media about the mpox outbreak.

### 3.5. Factors Associated with Scores of Knowledge, Attitude and Worry

We assessed the associated factors with a score of knowledge, attitude, and worry toward mpox; the results are presented in Table 2. The results of the independent sample *t*-test showed that there was a significant statistical difference between the mean scores of knowledge according to gender (*p* < 0.05) and marital status (*p* < 0.05). The analysis of variance showed statistically significant differences between the knowledge mean scores according to education level, and place of residence (*p* < 0.05). Tukey’s post hoc test results showed that mean scores of master’s and doctoral were higher than other levels of education (*p* < 0.05). Moreover, the findings of this test showed that the Sulaymaniyah participants’ mean scores of knowledge were higher than other participants from other cites (*p* < 0.05).

The results of the independent sample *t*-test showed that there was a significant statistical difference between the mean scores of attitudes according to gender (*p* < 0.05). The results of the analysis of variance showed that there were statistically significant differences between the mean scores of the attitude according to the level of education and place of residence (*p* < 0.05). The results of Tukey’s post-hoc test showed that there were significant differences between the mean scores of the attitudes of participants with bachelor’s and diploma degrees (*p* < 0.05). The findings of this test showed that there was a statistically significant difference between the mean scores of the attitude of people living in Sulaymaniyah and Duhok (*p* < 0.05).

The results of the independent sample *t*-test revealed that there were significant statistical differences between the mean scores of worry according to gender and marital status (*p* < 0.05). The mean score for worry was higher in married men. In addition, the results of this test showed that there were significant differences between the mean scores of worry of participants who had a bachelor’s degree with the ability to read and write and a diploma (*p* < 0.05). The results of this test showed that there were statistically significant differences between the mean scores of worry of participants living in Sulaymaniyah city, Erbil, and Duhok (*p* < 0.05).

### 3.6. Level of Knowledge, Attitude and Worry

We then classified the scores for knowledge, attitude, and worry into three groups: poor, fair, and good for knowledge; negative, neutral and positive for attitude; and low, moderate, and high for worry (Table 3). Our data revealed that 40.4% of the participants had a good level of knowledge about mpox. A majority (61%) of participants’ attitudes toward mpox were classified as negative. In addition, 31.0% of the present study participants had a high level of worry about mpox (Table 3). 

### 3.7. Logistic Regression Analysis to Evaluate of Predictors with the Level of Knowledge, Attitude, and Worry

As shown in Table 4, the univariate logistic regression revealed the factors that were significantly associated with good knowledge about mpox, which were: males compared with females (OR: 1.55), singles compared with married participants (OR: 2.12), participants that had the capability to write and read, secondary and high school, participants with a diploma degree compared to participants who had a master’s or doctoral degree (OR: 0.07, 0.13, and 022, respectively), and participants from Sulaymaniyah compared to the participants from Halabja (OR: 3.54). In addition, the results showed that the factors that were significantly associated with a positive attitude toward mpox, which were males compared with females (OR: 1.29), and participants from Sulaymaniyah compared with the participants from Halabja (OR: 2.83). Finally, the results showed the factors associated with a high level of worry were: males compared to females (OR: 3.04), single participants compared to married participants (OR: 0.61), Christian participants compared to other faiths participants (OR: 0.727), participants from Sulaymaniyah and Erbil compared to participants from Halabja (OR: 37.60 and 4.88, respectively).

In Table 5, the results of the multivariate logistic regression analysis showed that males had a higher chance of having good knowledge about mpox compared to females (aOR: 2.22; 95% CI: 1.32–3.73). Christian participants compared with the participants from the other faiths (aOR: 17.57; 95% CI: 1.17–262.63). All groups in the educational level had a better chance of having good knowledge about mpox compared to the participants with master’s and doctoral degrees (aOR: 0.05; 95% CI: 0.01–0.23; aOR: 0.11; 95% CI: 0.04–0.31; aOR: 0.16; 95% CI: 0.06–0.41; and aOR: 0.40; 95% CI: 0.16–0.99, respectively). Participants from Sulaymaniyah compared with the participants from Halabja (aOR: 3.20; 95% CI: 1.50–6.84). Moreover, the results showed that male participants had a greater chance of having a positive attitude toward mpox compared to the female participants (aOR: 2.28; 95% CI: 1.20–4.35). Participants from Sulaymaniyah had a better chance to have a positive attitude toward mpox compared to participants from Halabja (aOR: 2.67; 95% CI: 1.02–6.98). Finally, compared to female participants, male participants had a better chance of having a high level of worry toward mpox about thrice (aOR: 3.09; 95% CI: 1.73–5.51). Christian participants had a higher chance of having a high level of worry toward mpox compared to the participants in the other faiths (aOR: 0.02; 95% CI: 0.01–0.66). Participants who can write and read, and from the secondary and high school had better chance to have high level of worry toward mpox compared to the participants with master and doctoral (aOR: 0.16; 95% CI: 0.04–0.62, and aOR: 0.25; 95% CI: 0.09–0.66) respectively. Participants from Sulaymaniyah and Erbil had a better chance of having a high level of worry toward mpox compared to participants from Halabja (aOR: 37.83; 95% CI: 13.35–107.21 and aOR: 5.96; 95% CI: 2.02–17.55, respectively).

## 4. Discussion

The present study was conducted to assess the knowledge, attitude, and worry levels of the general population in the Kurdistan region of Iraq regarding the ongoing, multi-country mpox outbreak. Overall, the results revealed that the participants had a moderate level of mpox knowledge, a neutral attitude towards mpox, and a relatively moderate worry level.

The global burden of the COVID-19 pandemic has been felt over the past three years. The fact that it occurred during an era of widespread transportation networks and intercontinental contact and fast travelling means, in tandem with significant advancements in infection prevention measures compared to previous pandemics. Sophisticated levels of information technology, and telecommunication gave it a unique quality that set it apart from other pandemics in modern history [41].

Besides the obvious devastating health effects, the COVID-19 pandemic had widespread economic repercussions. Therefore, the announcement of the re-emergence of mpox, as well as its declaration of global health emergency and continuously rising cases, could have harmful effects on the societies regarding worry and anxiety, especially given the recent recovery of the international systems from the pandemic consequences and the loosening of the strict measures and restrictions that were applied on traveling, and personal communications. This worry could be related to the recent COVID-19 pandemic and the experience of people associated with it [42,43].

Epidemics and pandemics tend to occur at periodic intervals, i.e., they appear now and then, posing many challenges to different societies during these times. The lack of awareness frequently leads to a careless attitude, which can have an adverse effect on one’s capability to respond appropriately to these challenges [39]. The mental health of a community may be negatively influenced by the widespread effects of outbreaks and pandemics. Individuals in a community are influenced behaviorally as well as emotionally by the fear and worry that are associated with pandemics and epidemics [39]. As a result, the researchers here endeavored to assess people’s levels of knowledge, attitudes, and worry in Kurdish society of Iraq.

The results of the knowledge assessment questionnaire revealed participants’ knowledge gaps concerning MPXV infections, i.e., they had a moderate level of knowledge about mpox. Put another way, the respondents were not quite informed about the disease, its symptoms, causes, transmission, and treatment, as well as about how to handle people with the disease. The reason why people have this relatively limited amount of information regarding the disease can be associated with the fact that the disease has not yet been reported widely in the Middle East. Additionally, the participants did not have experience with the disease, and their knowledge was limited as knowledge of something that could partly stem from experience [27]. Based on previous research [28], the disease is a re-emerging disease in the world. Although the outbreak seems recent, knowledge of the disease by ordinary people can help prevent its development. Our results coincide with previous research not only among ordinary people, but among Jordanian medical, nursing, dentistry, and pharmacy students [27], Indonesian general practitioners [28], Saudi physicians with medical students [44], health professionals in Kuwait [45], and Italian physicians [33]. These groups of the human population are considered knowledgeable as they belong to the health sciences and should possess information at least regarding health-related topics. This highlights the recency of the disease, not in terms of its origin, but rather in terms of its reappearance. A dearth of knowledge concerning the transmission, treatment, and symptoms can negatively affect the control of the disease. Therefore, raising the population’s level of knowledge and awareness is suggested to successfully prevent the disease from spreading across the world.

Moving on to the variation in knowledge based on the socio-demographic characteristics, our study demonstrated that there were significant differences for gender, marital status, level of education, and place of residence on the knowledge scale that included questions about transmission, prevention, isolation, treatment, major symptoms, and source of information. Inconsistent with previous research [27,28], male participants in our study reported a higher level of knowledge than females. One explanation for this can be attributed to the dominant role that men have in society and that they are more or less responsible for the fatal issues being faced by the community. Furthermore, females frequently devote time and attention to make-up and cosmetics, as well as beauty gadgets, based on the researchers’ observation in the Kurdish society. Additionally, married participants reported a higher level of knowledge than unmarried participants, which seems both logical and reasonable. Because married people have more concerns about health issues and have more responsibility in society, they endeavor to acquire more knowledge. As for the level of education, people with master’s and doctoral degrees showed the highest amount of knowledge, followed by those with bachelor’s degrees, those with diploma degrees, those who could read and write, and then those who had a secondary school certificate. This result is rather confusing because the ranking order does not determine whether people with higher certificates show more knowledge or those with a lower level of education. Therefore, more research is needed to tackle the effect of education level on the amount of knowledge possessed by a certain population. The place of residence also produced the expected results; people living in the culturally and socially more developed province of Sulaymaniyah demonstrated a higher level of knowledge, followed by the Erbil, Duhok, and Halabja provinces. Contrary to our findings, previous research [27,32], detected no significant differences by place of residence. The distinction between the findings is related to the division of the variables into their components. For instance, the place of residence in our study includes the different provinces, while in [27], place of residence includes the division of the variable into someone being from the country or outside the country. Nevertheless, the concerning knowledge showed no significant difference with any of the socio-demographic variable.

The second principal variable of this study involved participants’ attitudes towards mpox, particularly towards vaccination, control of the disease, and travel related safety issues within the country. The results showed that the participants held neutral attitudes towards mpox. Although mpox has not spread among people, the attitudes were not reported to be positive. The rather low attitude of the participants indicates the adverse and preconceived effects of COVID-19 on this portion of the population. As Yoo states, “The wounds that the COVID-19 pandemic has inflicted on us are very deep and seemingly long-lasting and still affect us” [46]. Such attitudes revealed in this study contradict previous research [32,33], reporting positive attitudes because, unlike the participants of our study, these studies were conducted among physicians. Importantly, the limited knowledge of these study participants might be one reason behind the neutral attitudes expressed.

Participants’ attitudes toward mpox did not vary according to each of the demographic variable: age, religion, and marital status, i.e., participants showed similar attitudes towards mpox regardless of their age, religion, and marital status. However, their attitudes changed according to gender, education level, and place of residence. Males’ attitudes were more positive compared with females in terms of accepting people with the disease after recovery, receiving vaccination, travelling across the country, and controlling the disease. This finding supports and buttresses previous research which found that males were more positive in their attitudes [26,27]. Although Ricco et al. demonstrated the contrary [33], i.e., insignificant results. Furthermore, education level showed that people with a bachelor’s degree held more positive attitudes, while people with diplomas recorded the lowest mean score. The results for attitude, similar to the knowledge domain, produced confusing results. As for the place of residence, the results indicated that people from Sulaymaniyah province showed significantly more positive attitudes, followed by people from Halabja, Erbil, and Duhok. This might be associated with the adverse repercussions of COVID-19 that were more severe in Erbil, creating a sense of negativity toward pandemics.

However, another variable of the study involves worry that coincided with the knowledge domain and the attitude domain, i.e., participants reported a moderate level of worry, having problems regarding mpox in terms of thinking about the disease, feeling paranoid, avoiding social contact, speaking about mpox, having difficulties with sleeping, being affected by posts on social media about the pandemic, and related ideas. One source of worry about the pandemic originates from the media, which keeps people informed and has been reporting bad news. This creates lots of concern and fear for ordinary people, particularly those who have already been affected by COVID-19 [39]. As for the impact of the socio-demographic variables, the results showed significant differences for all of them, except for age. In other words, male participants were, to a greater degree, preoccupied with mpox. Furthermore, Muslims and people who had a bachelor’s degree, as well as people who belonged to the Sulaymaniyah governorate, were more worried as compared to non-Muslims, and people who had other degrees as well as people who belonged to other governorates, respectively.

Although mpox has not been reported in the current context and little is known about its adverse effects, not only general practitioners’ and physicians’ information about it can be useful, but ordinary people’s information about it can help prevent the disease from spreading through different regions. Additionally, having obtained information about mpox can enhance people’s attitudes, and this, in turn, might help them to eliminate their worry about the disease. 

## 5. Limitations

Although this study includes a large portion of the Kurdish population, it is restricted to people who have access to the Internet. People without Internet access may have significantly different knowledge, attitudes, and worries than those who do. More importantly, the study, considering its design in terms of data collection, is purely quantitative, using a questionnaire to fulfill its aims. The use of an interview might have unraveled the mysteries over mpox and provided more insight into the investigation of all three major variables of the study. Therefore, the findings of the study cannot be generalized to the whole population. In addition, one unreliable statement was excluded from the knowledge scale. As a result, the overall Cronbach’s alpha value increased significantly. Considering the setting where the study was conducted, it only included participants from the Kurdistan region located in the northern part of Iraq, which cannot represent people from other parts of Iraq due to cultural differences that are strongly associated with the knowledge, attitude, and worry of participants. As a result, future research might consider including participants from various parts of Iraq that may lead to different results.

## 6. Conclusions

In the current mpox outbreak, most educated people have sufficient knowledge of this viral infection which is urgently needed to raise public awareness and manage people’s emotional wellbeing. There has been no research conducted to date that assesses how the mpox epidemic affected people’s mental health. Researching the effects of mpox on people’s mental health in different groups—including the public; pregnant and lactating mothers; healthcare providers; immunocompromised patients; individuals infected with the virus; their close contacts; LGBTQ+ people; and people with pre-existing psychiatric and medical problems, who are at increased risk for adverse psychosocial outcomes—is crucial for developing efficient interventions for those affected. Since mpox can be transmitted from animals to humans, it is imperative that animal health officials also be involved in any and all prevention efforts, and one health approach to be strengthened and implemented widely to tackle zoonotic concerns of monkeypox. Considering the continuous rise in mpox cases in several countries and taking into mind any feasible pandemic probability of this disease amid the already ongoing COVID-19 pandemic, proactive control measures, adequate disease prevention strategies and preparedness plans need to be formulated and immediately implemented to tackle the appearance of fears and psychological disturbances among people and safeguard the mental health of the public. For this purpose, there is an urgent requirement to adopt suitable policies and recommendations to effectively prevent and control MPXV spread, as well as disseminate perfect scientific information related to public health concerns of this virus to explicitly counteract the infodemic’s misinformation and disinformation that could create confusion and unwanted panic and protect the mental health disturbances of the public.

## Figures and Tables

**Table 1:** Sociodemographic characteristics of the study sample (*n* = 510).

Variables	Items	Frequency	Percentage
Age	18–27	204	40.0
	28–37	186	36.5
	38–47	84	16.5
	48–57	36	7.0
Gender	Male	277	54.3
	Female	233	45.7
Marital status	Single	240	47.1
	Married	270	52.9
Religion	Islam	482	94.5
	Christian	23	4.5
	Other	5	1.0
Level of education	Able to read and write	31	6.0
	Secondary and high school	87	17.1
	Diploma	104	20.4
	Bachelor	204	40.0
	Master and doctoral	84	16.5
Residence area	Sulaymaniyah	255	50.0
	Erbil	118	23.0
	Duhok	70	13.8
	Halabja	67	13.2

**Table 2:** Factors associated with score of knowledge, attitude and worry toward mpox (*n* = 510).

Variables		Knowledge	Attitude	Worry
		Mean ± SD	Mean ± SD	Mean ± SD
Mean of Raw Score		6.86 ± 1.80	1.24 ± 1.08	32.38 ± 10.09
Sex	Male	7.1 ± 1.79	4.2 ± 1.6	34.6 ± 9.1
	Female	6.5 ± 1.77	3.8 ± 1.6	29.7 ± 10.5
*p* value		0.001	0.010	0.001
Age	18–27	6.6 ± 1.9	4.1 ± 1.7	32.2 ± 10.3
	28–37	6.9 ± 1.68	3.8 ± 1.5	33.3 ± 9.9
	38–47	7.0 ± 1.76	4.1 ± 1.7	31.4 ± 9.5
	48–57	7.3 ± 1.8	3.6 ± 1.2	29.9 ± 10.07
*p* value		0.116	0.139	0.195
Marital status	Single	6.5 ± 1.82	4.04 ± 1.6	31 ± 10.4
	Married	7.1 ± 1.74	4.01 ± 1.6	33.6 ± 9.6
*p* value		0.001	0.852	0.004
Religion	Islam	6.8 ± 1.8	4.05 ± 1.6	33 ± 9.8
	Christian	7.3 ± 1.55	3.3 ± 1.4	19.4 ± 4.4
	Other	6.0 ± 1.4	4.4 ± 0.89	32 ± 12.08
*p* value		0.283	0.145	0.001
Level of education	Able to read and write	5.9 ± 2.0	3.9 ± 1.7	29.9 ± 9.3
	Secondary and high school	6.0 ± 1.89	4.05 ± 1.7	29.3 ± 9.6
	Diploma	6.5 ± 1.67	3.5 ± 1.7	30.8 ± 10.5
	Bachelor	7.2 ± 1.72	4.2 ± 1.5	34.7 ± 9.4
	Master and doctoral	7.4 ± 1.5	4.03 ± 1.6	32.4 ± 10.5
*p* value		<0.001	0.011	0.001
Residence area	Sulaymaniyah	7.3 ± 1.74	4.3 ± 1.7	37.6 ± 7.6
	Erbil	6.5 ± 1.87	3.8 ± 1.4	30.5 ± 9.7
	Duhok	6.2 ± 1.51	3.3 ± 1.3	24 ± 8.7
	Halabja	6.3 ± 1.78	3.8 ± 1.4	24.1 ± 7.4
*p* value		<0.001	0.001	0.001

**Table 3:** Identifying knowledge, attitude and worry of the participants toward mpox epidemic (*n* = 510).

	Knowledge		Attitude		Worry	
	Frequency (%)	Mean SD	Frequency (%)	Mean SD	Frequency (%)	Mean SD
Poor/negative/low	130 (25.5)	6.86 ± 1.80	311 (61.0)	1.24 ± 1.08	176 (34.5)	32.38 ± 10.09
Fair/neutral/moderate	174 (34.1)		137 (26.9)		176 (34.5)	
Good/positive/high	206 (40.4)		62 (12.2)		158 (31.0)	
Result	Moderate level of knowledge toward mpox		Neutral attitude level toward mpox		Having moderate level of worry about mpox	

**Table 4:** Univariate logistic regression analysis to evaluate the predictive factors associated high knowledge, attitude and worry scores toward mpox (*n* = 510).

Items	Good Knowledge				Positive Attitude				High Level of Worry			
	OR	95% CI		*p*	OR	95% CI		*p*	OR	95% CI		*p*
Age in years (48–57 vs. others)												
18–27	0.40	0.16	1.01	0.053	7.26	0.94	55.65	0.056	1.26	0.51	3.07	0.608
28–37	0.50	0.20	1.28	0.151	3.32	0.42	26.21	0.254	1.32	0.54	3.23	0.542
38–47	0.65	0.23	1.83	0.420	7.42	0.92	59.68	0.059	0.71	0.25	1.96	0.513
Gender (Female vs. male)												
Male	2.12	1.35	3.32	<0.01	2.33	1.29	4.22	<0.01	3.04	1.94	4.76	<0.01
Marital status (Married vs. single)												
Single	0.50	0.32	0.78	<0.01	1.44	0.83	2.49	0.188	0.61	0.40	0.95	0.029
Religion (other faiths vs. others)												
Muslim	4.64	0.47	45.16	0.186	0.65	0.10	3.96	0.646	0.64	0.05	6.29	0.565
Christian	13.00	0.97	172.94	0.052	0.70	0.09	5.18	0.727	0.01	0.00	0.51	0.023
Educational level (Master or doctoral vs. others)												
Can write and read	0.07	0.02	0.27	<0.01	1.37	0.36	5.21	0.643	0.47	0.16	1.41	0.182
Secondary and high school	0.13	0.05	0.33	<0.01	1.27	0.46	3.55	0.637	0.51	0.23	1.10	0.086
Diploma	0.22	0.09	0.54	<0.01	0.88	0.30	2.52	0.815	0.82	0.40	1.64	0.577
Bachelor	0.48	0.21	1.08	0.077	1.86	0.76	4.52	0.168	1.65	0.88	3.10	0.116
Residence (Halabja vs. others)												
Sulaymaniyah	3.54	1.78	7.03	0.019	2.83	1.13	7.05	0.025	37.60	13.76	102.72	<0.01
Erbil	1.29	0.61	2.72	0.492	0.71	0.22	2.26	0.572	4.88	1.73	13.78	<0.01
Duhok	0.76	0.32	1.82	0.549	0.43	0.10	1.84	0.259	1.34	0.39	4.52	0.635

OR: odds ratio; CI: confidence interval; vs.: versus.

**Table 5:** Multivariate logistic regression analysis to evaluate the predictive factors associated high knowledge, attitude, and worry scores toward mpox (*n* = 510).

Items	Good Knowledge				Positive Attitude				High Level of Worry			
	aOR	95% CI		*p*	aOR	95% CI		*p*	aOR	95% CI		*p*
Age in years (48–57 vs. others)												
18–27	0.65	0.21	2.01	0.458	7.19	0.84	61.18	-	-	-	-	-
28–37	0.42	0.14	1.23	0.117	3.74	0.46	30.28	-	-	-	-	-
38–47	0.38	0.12	1.23	0.110	8.17	0.99	67.20	-	-	-	-	-
Gender (Female vs. male)												
Male	2.22	1.32	3.73	<0.01	2.28	1.20	4.35	0.012	2.60	1.49	4.54	<0.01
Marital status (Married vs. single)												
Single	0.65	0.34	1.24	0.200	1.55	0.70	3.42	0.270	1.24	0.69	2.22	0.469
Religion (other faiths vs. others)												
Muslim	2.56	0.24	27.36	0.436	-	-	-	-	0.35	0.02	4.59	0.42
Christian	17.57	1.17	262.63	0.038	-	-	-	-	0.02	0.01	0.66	0.02
Educational level (Master or doctoral vs. others)												
Can write and read	0.05	0.01	0.23	<0.01	0.78	0.18	3.31	0.745	0.16	0.04	0.62	<0.01
Secondary and high school	0.11	0.04	0.31	<0.01	0.72	0.22	2.28	0.577	0.25	0.09	0.66	<0.01
Diploma	0.16	0.06	0.41	<0.01	0.56	0.18	1.73	0.320	0.42	0.17	1.05	0.064
Bachelor	0.40	0.16	0.99	0.048	1.08	0.40	2.88	0.868	0.77	0.34	1.77	0.552
Residence (Halabja vs. others)												
Sulaymaniyah	3.20	1.50	6.84	<0.01	2.67	1.02	6.98	0.044	37.83	13.35	107.21	<0.01
Erbil	1.07	0.47	2.45	0.862	0.77	0.23	2.51	0.670	5.96	2.02	17.55	<0.01
Duhok	0.59	0.22	1.54	0.283	0.47	0.11	2.03	0.315	1.79	0.51	6.26	0.362

aOR: adjusted odds ratio; CI: confidence interval; vs.: versus.

## Data Availability

Data is available from the corresponding author upon reasonable request.

## Supplementary Materials

The following supporting information can be downloaded at: https://www.mdpi.com/article/10.3390/vaccines11030610/s1, Supplementary File S1: Knowledge, Attitude and Worry in the Kurdistan Region of Iraq During the Mpox (Monkeypox) Outbreak in 2022: An Online Cross-sectional Study.
