# Supplementary material for: Knowledge, Attitude and Worry in the Kurdistan Region of Iraq during the Mpox (Monkeypox) Outbreak in 2022: An Online Cross-Sectional Study
Source: Vaccines (Basel). 2023 Mar 8;11(3):610. doi: 10.3390/vaccines11030610 (PMC10054073; doi:10.3390/vaccines11030610)
Supplement: Supplementary file 1 [file vaccines-11-00610-s001.zip › vaccines-2216929-supplementary.pdf]

Knowledge, Attitude and Worry in the Kurdistan Region of Iraq During the Mpox (Monkeypox) Outbreak in 2022: An Online Cross-sectional Study

Consent question: -

This survey aims to assess the knowledge, attitude, and worry about monkeypox among general population of Kurdistan Region. You are then kindly invited to answer the following online survey (on your computer or your mobile phone). We would like to kindly ask you to do your best to answer all questions, if possible, because this will allow us to anonymously process and analyze the data, and thus, improve the knowledge in the field.

Part 1: Socio-demographic characteristics.

Characteristics	Participants N (%)
Age in years	
Gender	
Male	
Female	
Marital status	
Single	
Married	
Others	
Religion	
Islam	
Christian	
Others	
Level of Education	
Ability to read and/or write	
Middle/High School	
Diploma	
Bachelor	
Master and above	
Geographic distribution	
Sulaimani	
Erbil	
Duhok	
Halabja	

Part 2: Knowledge of participants about monkeypox epidemic (Three Likert scale and checkboxes questionnaire).

Questions	True	False	I don’t know
1. Case isolating a person with symptoms stop the spread of the monkeypox virus?	True	False	I don’t know
2. Pets at the home able to transmit monkeypox virus?	True	False	I don’t know
3. Monkeypox is a viral disease infection?	Yes	No	
4. Before 2022 have you heard about monkeypox virus infection	Yes	No	
5. Attended lectures/discussions about monkeypox virus infection	Yes	No	

6. MPX may be transmitted through
Touching
Sneezing
Sexual intercourse
Food
All the above
7. Symptoms of monkeypox infection are
Fever
Headache
Muscle aches and backache
Chills
Exhaustion
Swollen lymph nodes
Skin rash
8. What is you source information about monkeypox virus infection?
Social media
Television and news papers
friends

Part 3: Attitude of participants about monkeypox epidemic (Three-Likert scale).

Questions	Yes	No	I don't know
1. Patients with monkeypox infection, who are declared cured should not be allowed to stay within the community at this time?			
2. If made available, would you receive monkeypox vaccine?			
3. Do you think traveling across/within the country is safe during these times?			
4. Do you agree that the monkeypox will finally be successfully controlled?			

Part 4: Worry related to monkeypox outbreak in 2022 (Five-Likert scale).

Over the last 2 weeks, how often have you been bothered by the following problems?	Never	Rarely	sometimes	Often	Always
1. From the last 2 weeks how often do you think about monkeypox outbreak?					
2. From the last 2 weeks how often you feel paranoid about contacting monkeypox virus infection?					
3. From the last 2 weeks how, often you avoid social contact?					
4. From the last 2 weeks, how often you have talked to your friends about the monkeypox epidemic?					
5. From the last 2 weeks, how often you have had difficulty sleeping by being worried about the monkeypox epidemic?					
6. From the last 2 weeks, how often you feel affected by the posts on social media about monkeypox virus infection?					
7. From the last 2 weeks, how often do you feel worried about yourself, and close ones regarding the spread of monkeypox viral Infection?					
8. From the last 2 weeks, how often does the Idea of monkeypox viral Infection freak you out leading to inappropriate behaviours with anyone?					
9. From the last 2 weeks, how often does the Idea of monkeypox viral Infection freak you out post on social media?					

References

28. Harapan, H.; Setiawan, A.M.; Yufika, A.; Anwar, S.; Wahyuni, S.; Asrizal, F.W.; Sufri, M.R.; Putra, R.P.; Wijayanti, N.P.; Salwiyadi, S. Knowledge of Human Monkeypox Viral Infection among General Practitioners: A Cross-Sectional Study in Indonesia. *Pathog. Glob. Health* **2020**, *114*, 68–75.
33. Riccò, M.; Ferraro, P.; Camisa, V.; Satta, E.; Zaniboni, A.; Ranzieri, S.; Baldassarre, A.; Zaffina, S.; Marchesi, F. When a Neglected Tropical Disease Goes Global: Knowledge, Attitudes and Practices of Italian Physicians towards Monkeypox, Preliminary Results. *Trop. Med. Infect. Dis.* **2022**, *7*, 135.
39. Roy, D.; Tripathy, S.; Kar, S.K.; Sharma, N.; Verma, S.K.; Kaushal, V. Study of Knowledge, Attitude, Anxiety & Perceived Mental Healthcare Need in Indian Population during COVID-19 Pandemic. *Asian J. Psychiatr.* **2020**, *51*, 102083.
